# Phenotypic heterogeneity unveils a negative correlation between antibiotic resistance and quorum sensing in *Pseudomonas aeruginosa* clinical isolates

**DOI:** 10.3389/fmicb.2024.1327675

**Published:** 2024-02-12

**Authors:** Xiting Yang, Qianglin Zeng, Shiyi Gou, Yi Wu, Xiaoling Ma, Hang Zou, Kelei Zhao

**Affiliations:** ^1^Antibiotics Research and Re-evaluation Key Laboratory of Sichuan Province, School of Pharmacy, Chengdu University, Chengdu, Sichuan, China; ^2^Affiliated Hospital of Chengdu University, Chengdu University, Chengdu, Sichuan, China

**Keywords:** *Pseudomonas aeruginosa*, COPD, quorum-sensing, antibiotic resistance, mutation, evolution

## Abstract

Colonization of *Pseudomonas aeruginosa* in the lung environments frequently leads to the enrichment of strains displaying enhanced antibiotic resistance and reduced production of quorum-sensing (QS) controlled products. However, the relationship between the emergence of QS deficient variants and antibiotic resistance remains less understood. In this study, 67 *P. aeruginosa* strains were isolated from the lungs of 14 patients with chronic obstructive pulmonary disease, followed by determining their genetic relationship, QS-related phenotypes and resistance to commonly used antibiotics. The integrity of *P. aeruginosa* QS system was checked by DNA sequencing. The relationship between the QS system and antibiotic resistance was then assessed by correlation analyses. The function of the LasR protein and bacterial virulence were evaluated through homology modeling and nematode-infection assay. The influence of antibiotic on the development of extracellular protease production ability of *P. aeruginosa* was tested by an evolutionary experiment. The results showed that *P. aeruginosa* clinical strains displayed abundant diversity in phenotype and genotype. The production of extracellular proteases was significantly negatively correlated with antibiotic resistance. The strains with enhanced antibiotic resistance also showed a notable overlap with the mutation of *lasR* gene, which is the core regulatory gene of *P. aeruginosa* QS system. Molecular docking and *Caenorhabditis elegans* infection assays further suggested that *P. aeruginosa* with impaired LasR protein could also have varying pathogenicity. Moreover, *in vitro* evolution experiments demonstrated that antibiotic-mediated selective pressure, particularly from Levofloxacin contributed to the emergence of extracellular protease-negative strains. Therefore, this study provides evidence for the connection of *P. aeruginosa* QS system and antibiotic resistance, and holds significance for developing targeted strategies to address antibiotic resistance and improving the management of antibiotic-resistant infections in chronic respiratory diseases.

## Introduction

Multidrug resistance (MDR) has been increased all over the world that is considered a public health threat (Magiorakos et al., 2012; Algammal et al., 2023a). Several recent investigations reported the emergence of multidrug-resistant bacterial pathogens from different origins that increase the necessity for the proper use of antibiotics (Algammal et al., 2022, 2023b; Elbehiry et al., 2022; Shafiq et al., 2022). The common and essential use of antibiotics in managing chronic bacterial infections, often involving concurrent administration of multiple antibiotics, inevitably leads to the emergence and proliferation of MDR bacterial strains.

*Pseudomonas aeruginosa* is a predominant pathogen in hospital-associated MDR events (King, 2019; Mac Aogáin et al., 2021). It can cause various infections, serving as a major contributor to sepsis and neutropenia in immunocompromised patients, and standing as a primary cause of hospital-acquired pneumonia and respiratory failure (Gallego et al., 2014; Rodrigo-Troyano et al., 2018). *Pseudomonas aeruginosa* is one of the most frequently isolated bacterial pathogens from airway samples (Garcia-Vidal et al., 2009). It poses a significant challenge as it secretes various virulence factors, such as elastase and lipase, which can destroy lung tissue and aggravate inflammatory reactions. Additionally, *P. aeruginosa* can form biofilms, which can resist elimination by the host immune system and inhibit the effects of antibiotics (Filloux, 2011; Hilker et al., 2015). Therefore, regular antimicrobial susceptibility testing is essential for selecting appropriate antibiotics and detecting emerging multidrug-resistant strains of *P. aeruginosa*. Simultaneously, a thorough investigation into the characteristics and pathogenicity of clinical *P. aeruginosa* is crucial for identifying and evaluating infection control strategies (Høiby et al., 2015; Miravitlles and Anzueto, 2017; Behzadi et al., 2021; Langendonk et al., 2021).

The quorum-sensing (QS) system of *P. aeruginosa* is known to be involved in cell-to-cell communication mediated by signaling molecules that respond to changes in cell density (Schuster and Greenberg, 2006; Jimenez et al., 2012). The essential communication facilitated by QS enables coordinated behavior and regulates key virulence factors in *P. aeruginosa*, enhancing pathogenicity and environmental adaptability (Wilder et al., 2011; Chevalier et al., 2017). For instance, pyocyanin, a phenazine, aids in redox reactions for interspecies competition; proteases facilitate tissue invasion and immune evasion; rhamnolipids, acting as surfactants, contribute to adhesion, biofilm formation, and migration (Hancock and Speert, 2000; Liang et al., 2014). Additionally, QS-controlled motility (swimming, swarming, and twitching) crucially influences biofilm formation and increases antibiotic resistance (Oshri et al., 2018; Pena et al., 2019). Currently, three QS systems, including *las, rhl*, and *pqs*, have been confirmed in *P. aeruginosa*. LasR, RhlR, and PqsR are three transcriptional regulatory proteins at the core of the interlinked QS system, interacting with homologous chemical molecules to induce the expression of downstream virulence factors (Kostylev et al., 2019).

Generally, the virulence factors of *P. aeruginosa* are not exclusively regulated by a single QS pathway. However, clinical experience suggests that *P. aeruginosa* in chronic respiratory infections exhibits phenotypic heterogeneity in cell motility and the production of characteristic virulence factors, indicating variations in QS system integrity (Heurlier et al., 2006; Feliziani et al., 2014; Jiricny et al., 2014; Vanderwoude et al., 2020). Previous research has indicated that this heterogeneity is influenced by environmental stress and the adaptability of *P. aeruginosa*, manifested in dissolved oxygen levels inside and outside the biofilm and low nutrient concentrations (Smith et al., 2006; Jorth et al., 2015; Zhao et al., 2016; Lozano et al., 2018; Figueiredo et al., 2021; Grekov et al., 2021). Theoretically, environmental pressures on the QS system, including antibiotics, may partially influence the rise in bacterial resistance. LasR mutants of *P. aeruginosa* isolated from cystic fibrosis patients exhibit a distinct QS regulatory hierarchy compared to the wild-type (Feltner et al., 2016). However, it is unclear if environmentally adaptive QS variants may be associated with clinical multidrug-resistant events.

Therefore, this study aims to explore the association between QS-related phenotypes and antibiotic resistance of *P. aeruginosa* clinical strains, with the expectation of providing valuable insights into the identification of clinical infection types and the application of antibiotic management. Firstly, we isolated 67 *P. aeruginosa* strains from the respiratory samples of 14 patients with chronic obstructive pulmonary disease (COPD). Subsequently, 26 representative strains were phenotypically identified through assays involving extracellular proteases, pyocyanin, biofilm, and cell motilities. Further analyses, including antimicrobial susceptibility testing, gene sequencing, and correlation assessments, revealed a connection between *lasR* mutations and phenotypic variations as well as antibiotic resistance. The functionality of the LasR protein and overall bacterial virulence were assessed through homology modeling and nematode infection assay. Finally, an evolutionary experiment simulated the impact of selective pressure from a single antibiotic on the QS system and the production of extracellular proteases.

## Materials and methods

### Ethics statement

Bronchoalveolar lavage fluids and sputum samples were obtained from 14 COPD patients hospitalized in the Department of Respiratory and Critical Care Medicine at the affiliated hospital of Chengdu University (Supplementary Table S1). Written informed consent was obtained from the patients or their immediate family members, and the study was approved by the Ethics Committee of the Affiliated Hospital of Chengdu University (Approval Code: PJ2020-021-03).

### Identification of *P. aeruginosa* from clinical samples

The respiratory samples were stored on ice, liquefied by adding an equal volume of phosphate buffer solution (PBS), and cultured overnight at 37°C on lysogeny broth (LB; Hope Bio-Technology Co., Ltd, Qingdao, China) plates. Colonies with discernible variations in size, color, and surface smoothness were chosen and were subjected to 16S rRNA sequence-based species identification.

### Phenotypic identification

Phenotypic characterization of *P. aeruginosa* COPD isolates was conducted using the methods described elsewhere (Zhao et al., 2020). Chemical reagents for the following experiments were purchased from Chengdu Kelong Chemical Co., Ltd. (Chengdu, China) unless specified otherwise. All the experiments were independently repeated three times and compared to the values of the reference strain wild-type (WT) PAO1, which was preserved in our laboratory (Zhao et al., 2020). In brief, the production of pyocyanin was determined by measuring the optical density at 520 nm (OD_520_) of the liquid after HCl-chloroform extraction. Biofilm production was assessed by staining with 0.1% ammonium oxalate-crystal violet dye, followed by elution with 95% ethanol. The eluate's optical density at 595 nm (OD_595_) was measured. For the cell motility assay, the bacterial solution is inoculated in the center of an LB plate with 0.5% agar and in the bottom plastic layer of an LB plate with 1% agar, followed by measuring the colony movement diameter. For the production of extracellular proteases, inoculate bacteria on a 0.5% M9-skim milk (Sigma-Aldrich, USA) plate and measure the protease hydrolysis zone's diameter. For the adenosine utilization assay, inoculate bacterial solution on the surface of M9 solid medium with 0.1% adenosine (Sigma-Aldrich, USA) and assess colony growth relative to WT PAO1. Growth equivalent to WT PAO1 is denoted as +, better than WT PAO1 as ++, worse than WT PAO1 as –, and no growth as null. For the mucoid transformation level assay, mucus transformation was evaluated by observing colony growth on LB plates after 24 h of incubation at 37°C. The results were categorized as follows: null for round colonies without mucus between adjacent colonies, – for round colonies with adjacent mucus, and + for deformed, liquid colonies with viscous adjacent colonies.

### Enterobacterial repetitive intergenic consensus-polymerase chain reaction

Enterobacterial repetitive intergenic consensus-polymerase chain reaction (ERIC-PCR) is a reliable method for analyzing genomic structure and genetic relationships among various bacteria, even non-enterobacteria (Stehling et al., 2010). Genomic DNA from clinical strains of *P. aeruginosa* and WT PAO1 was extracted using a Bacterial DNA Isolation Kit (Foregene Biotechnology, Co. Ltd., China), followed by PCR amplification using the single primer 5′-AAGTAAGTGACTGGGGTGAGCG-3′. Images were captured and processed using Image J v1.53a software. To construct a dendrogram, a binary matrix was created by designating the presence and absence of bands as 1 and 0, respectively, at the same positions. The matrix was then subjected to clustering analysis using the Cluster Vis platform at https://biit.cs.ut.e/ClustVis/.

### Antimicrobial susceptibility testing

The minimum inhibitory concentrations (MICs) of commonly prescribed clinical antibiotics, including aminoglycosides (Amikacin, Gentamicin, and Tobramycin), antipseudomonal carbapenems (Imipenem), antipseudomonal cephalosporins (Cefotaxime, Cefepime), antipseudomonal fluoroquinolones (Levofloxacin, Ciprofloxacin), monobactams (Aztreonam), and polymyxins (Polymyxin B), were determined against *P. aeruginosa* isolates using the broth microdilution method according to the guidelines of the Clinical and Laboratory Standards Institute (CLSI, 2022). Ciprofloxacin and Levofloxacin were purchased from Shanghai Titan Technology Co., Ltd., and other antibiotics were purchased from Shanghai YuanYe Biotechnology Co., Ltd. The multiple antibiotic resistance (MAR) index was calculated following the method established by Krumperman (1983). Subsequently, the MAR index was categorized into high and low based on its median value. MAR values below the median were classified as low, while values equal to or exceeding the median were designated as high (Puspita et al., 2021).

### PCR amplification

The complete sequences of the core regulatory genes of the *P. aeruginosa* QS system were initially amplified using PCR (*lasR*: 5′-TCAAACGCTGCGGTCTATT-3′ and 5′-CATCTCGCCCAGCAGTTT-3′, *lasI*: 5′-GCAGGGTTCTCGCCA TTCT-3′ and 5′-GCACCACCCACAGCATC-3, *rhlR*: 5′-GCTG GCATAACAGATAGGGT-3′ and 5′-CTCTCAGTCGGAGGACA TAC-3′, *rhlI*: 5′-ATCCGATGCTGATGTCCAA-3′ and 5′-TCTTCCGTGCGGTAGCTG-3′, *pqsR*: 5′-TTTCTTAGAACCGTT CCTGG-3′ and 5′-TGCTGGAGAACGCTCTACTC-3′), followed by DNA sequencing and sequence alignment to the genes of WT PAO1 (NCBI accession number: AE004091.2).

### Homology modeling and molecular docking

We employed a multi-step approach that integrates homology modeling and molecular docking to investigate the binding interactions between LasR mutant variants and self-inducing ligands. Initially, the *lasR* gene sequences of seven distinct *lasR* mutants of *P. aeruginosa* COPD isolates in the present study, encompassing both point mutations and deletions, were modeled. To generate accurate 3D structures, we utilized the Swiss-Model (https://swissmodel.expasy.org/) and AlphaFold (https://alphafold.com/) platforms. Structural completion, energy minimization, and site-directed mutagenesis were conducted using SPDBV v4.10 software. Subsequently, we evaluated the structural disparities among the LasR mutant models to pinpoint unique features associated with each variant. Following model generation, we conducted molecular docking experiments with Dockey v0.8.2 (Du et al., 2023). Twenty docking runs were performed for each LasR mutant with self-inducing ligands to evaluate differences in binding free energies and interaction sites. PyMOL facilitated the visualization and analysis of results, offering insights into structural and functional distinctions among LasR variants.

### *Caenorhabditis elegans* infection models

*Caenorhabditis elegans* serves as a valuable model organism for investigating the interplay between bacterial virulence systems and host defense mechanisms (Balasubramanian et al., 2016). For the fast-killing assay, we used the Peptone-Glucose-Sorbitol medium, while Nematode Growth Medium was employed for slow-killing assay. Nematodes were cultured at 37°C for 24 h. We introduced ten nematodes (for acute infection) or fifteen nematodes (for chronic infection), synchronized to the L4 stage, to plates with various bacterial strains and maintained them in a 25°C incubator. We monitored nematode survival at 4-h intervals for acute infection and 8-h intervals for chronic infection. Each experimental group included control groups fed with *Escherichia coli* OP50, a non-pathogenic strain commonly used to support nematode growth and reproduction.

### *In vitro* evolution experiment under antibiotic stress

The evolution of WT PAO1 and its isogenic *lasR* mutant, PAO1-Δ*lasR*, in which the *lasR* gene was replaced by gentamicin cassette as described elsewhere (Zhao et al., 2019), under antibiotic pressure, was determined by using the methods established by Hernando-Amado et al. (2019) with slight modifications. Initially, the MICs of Polymyxin B, Cefepime, and Levofloxacin for WT PAO1 and PAO1-Δ*lasR* were determined (Supplementary Table S2). Sub-MIC concentrations were established by appropriately reducing antibiotic concentrations in LB broth, where growth rates were inhibited but viability remained unaffected. The initial antibiotic concentrations were as follows: Polymyxin B at 1.5 μg/ml, Cefepime at 1.5 μg/ml, and Levofloxacin at 0.25 μg/ml for WT PAO1. For PAO1-Δ*lasR*, the initial antibiotic concentrations were Polymyxin B at 0.25 μg/ml, Cefepime at 1 μg/ml, and Levofloxacin at 0.25 μg/ml. In the evolutionary experiment, WT PAO1 and PAO1-Δ*lasR* were cultured in 2 ml deep 96-well plates containing 1 ml of LB broth and Polymyxin B, Cefepime, or Levofloxacin at sub-MICs at 37°C with shaking (180 rpm), respectively. Subsequently, 100 μl of the culture liquid was transferred to fresh media with higher antibiotic concentrations every 3 days. The antibiotic concentrations were progressively increased until reaching final levels of Polymyxin B at 32 μg/ml, Cefepime at 128 μg/ml, and Levofloxacin at 32 μg/ml (Supplementary Table S3). The resulting cultures were diluted and plated on LB agar plates, from which 10 individual colonies were randomly selected per plate, yielding a total of 100 colonies per experimental group. Extracellular protease production was assessed and the *lasR* gene of the WT PAO1 group's cultured products was sequenced to investigate the genetic changes.

### Statistical analysis

Statistical analysis was primarily conducted using GraphPad Prism v9.0. Quantitative experiments related to phenotypes were subjected to *t*-tests, and correlation analysis between drug resistance and phenotype was performed by calculating Spearman's rank correlation coefficient. Survival curves of nematodes were analyzed using the Log-rank (Mantel-Cox) test. The heatmaps for antibiotic resistance and bicomponent correlation analysis were generated using the online software package ChiPlot (https://www.chiplot.online/, Ji and Tang, 2022; Li et al., 2023). Molecular docking analysis was performed using Dockey and PyMOL software. Custom Python scripts were utilized for some composition ratio statistical analyses.

## Results

### COPD-derived *P. aeruginosa* strains display diversity in phenotype and genotype

We quantitatively or qualitatively measured QS-related phenotypes in clinically isolated *P. aeruginosa* strains, primarily to trace genetic and phenotypic differences among infection-causing strains, reflecting their diverse characteristics. The results of phenotypic identification revealed that about 80% (56/67) of *P. aeruginosa* clinical strains lost the capacity to produce extracellular proteases (Supplementary Figure S1). Moreover, 22% (15/67) of strains surpassed PAO1 in pyocyanin production, while 42% (28/67) produced less. In terms of biofilm production, 27% (18/67) exhibited a significant increase over PAO1, and 18% (12/67) showed a notable decrease. Regarding motility, 13% (9/67) had enhanced swarming compared to PAO1, while 40% (27/67) displayed reduced motility. In twitching motility, 9% (6/67) exceeded PAO1, and 43% (29/27) fell short.

**Figure 1:**
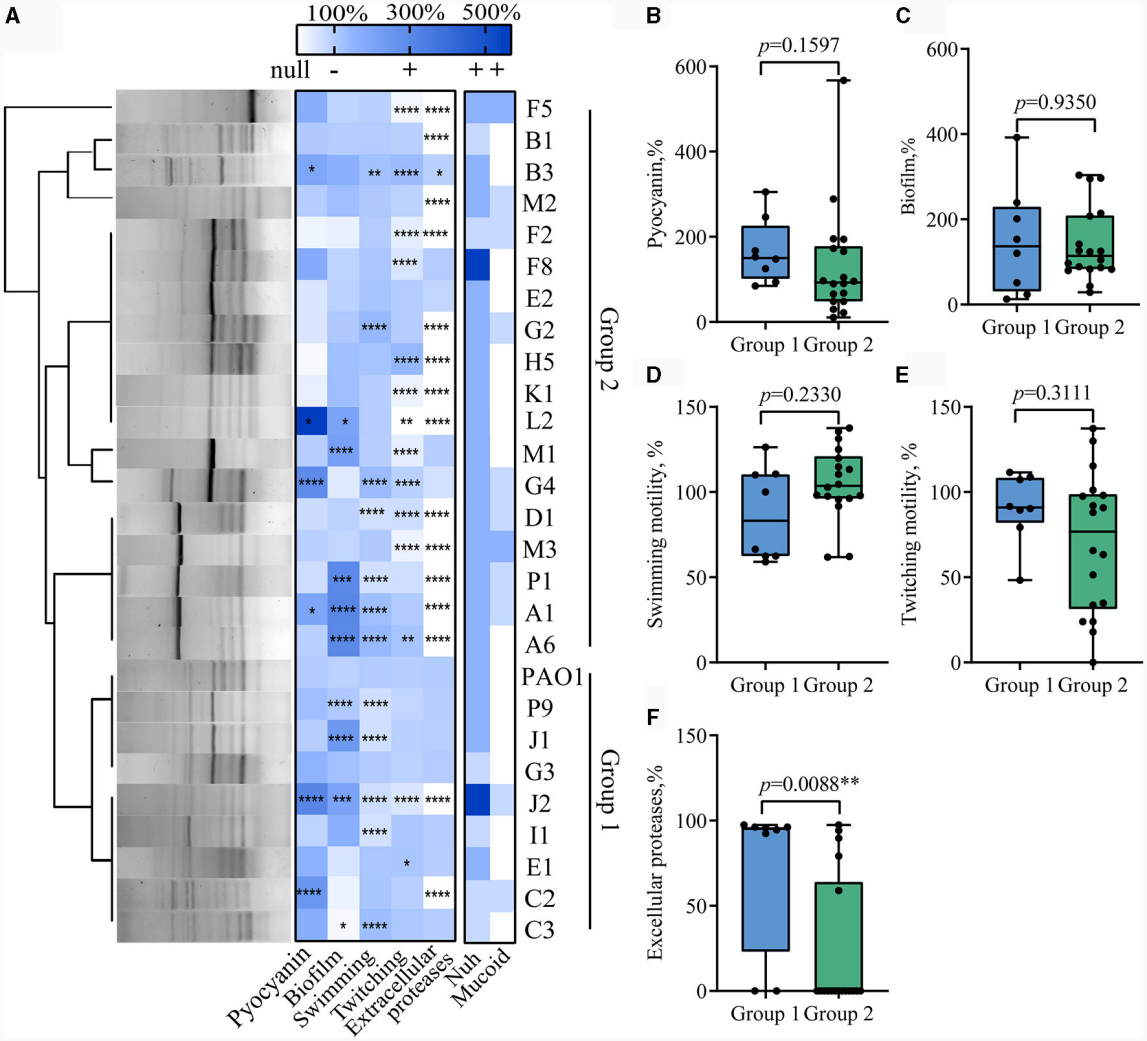
Diversity analysis of QS-related virulence phenotypes in clinical *Pseudomonas aeruginosa* isolates. **(A)** Genomic fingerprint typing and phenotypic thermography of the clinical *P. aeruginosa* isolates. The left side of the figure is the gel map of Eric PCR products after clustering, and the right side is the heat map of phenotypes related to the regulation of the QS system. *t*-test, Data are mean ± SD, *n* = 3 per group. **(B–F)** Box plots comparing extracellular proteases, pyocyanin, biofilm production, swimming motility, and twitching motility between Group 1 (*n* = 8) and Group 2 (*n* = 18). *p < 0.05, **p < 0.01, ***p < 0.001, ****p < 0.0001.

Subsequently, 26 strains exhibiting distinct phenotypic variations (including colony shape, color, size, and surface characteristics) were chosen for further investigation. The levels of pyocyanin and biofilm production in *P. aeruginosa* from the same sample exhibited significant fluctuations. Genomic DNA-based fingerprinting was utilized to investigate the genetic relationships among the strains. Cluster analysis of the gel electrophoresis bands generated via ERIC-PCR identified 10 different types of *P. aeruginosa*, with four of them detected in various patient samples (Figure 1). The combination of phenotype identification and genotypic typing allows the selected clinical strains to be classified into two groups. The first group comprised 8 strains that exhibited genotypic similarity to WT PAO1. These strains, except C2 and J2, demonstrated positive extracellular protease production. In contrast, the second group displayed significant genotypic dissimilarity from WT PAO1 and exhibited negative extracellular protease production (except for strains B3, F8, E2, M1, and G4).

Notably, even the strains with close genetic relationships isolated from the same patient displayed significant phenotypic differences and thus indicated substantial phenotypic heterogeneity among clinical strains. For instance, J1 and J2, despite having over 85% similarity in banding patterns, displayed distinct extracellular protease production capabilities, with J2 completely losing its extracellular protease production ability, while J1 retained the capacity to produce extracellular proteases similar to WT PAO1. Similar instances were observed with C2 and C3, B1 and B3, and F2 and F8 also.

### *Pseudomonas aeruginosa* COPD isolates have a complex antibiotic resistance spectrum

To assess the antibiotic resistance of these clinical *P. aeruginosa* strains, in conjunction with the types of antibiotics administered to patients during hospitalization (Supplementary Table S1), this study selected 10 commonly used antibiotics and determined the MICs of the strains against these antibiotics. Antibiotic susceptibility tests indicated that the majority of *P. aeruginosa* clinical isolates in this study exhibited lower susceptibility to the tested antibiotics compared to the WT PAO1 (Supplementary Table S4). Among the 26 isolated *P. aeruginosa* strains, over half exhibited intermediate or resistant profiles to commonly used antibiotics for respiratory tract infection, such as Cefotaxime (54%, *n* = 14), Imipenem (54%, *n* = 14), and Aztreonam (50%, *n* = 13). Approximately 4%−15% of strains exhibit resistance to Tobramycin, Amikacin, Levofloxacin, and Polymyxin B.

**Figure 2:**
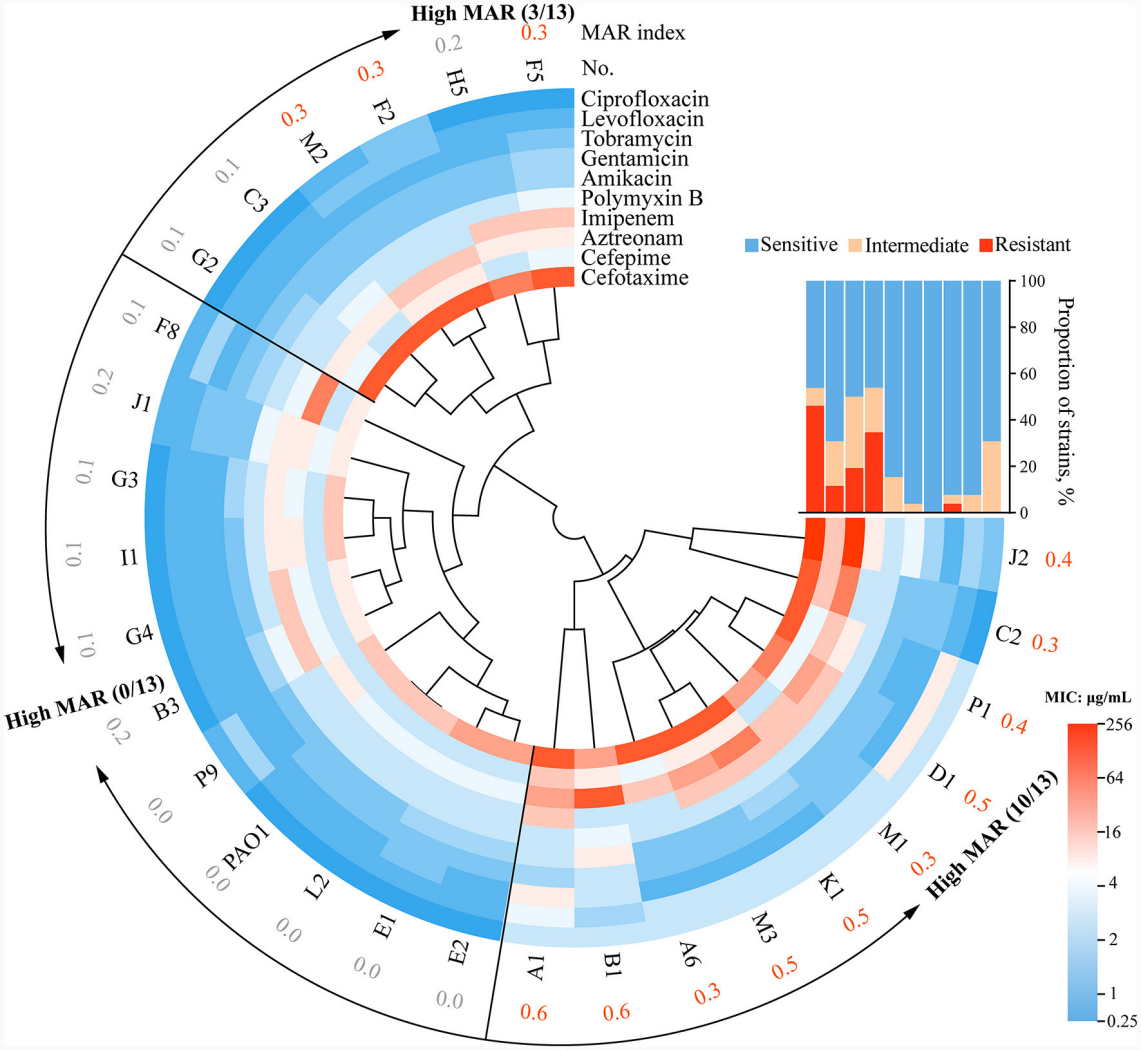
The circle diagram of drug resistance typing of clinical strains and the sensitivity rates to 10 antibiotics. Twenty-seven strains, including WT PAO1, were split into three groups based on the clustering results. MAR index = (total number of antibiotics tested)/(number of antibiotics resistant). Specifically, strains with a MAR index < 0.3 were classified as low MAR, while those with a MAR index equal to or >0.3 were classified as high MAR, with the classification based on the median MAR index. The strains assigned the orange code based on the corresponding MAR index are defined as high MAR Group in this study. The interpretation scope is referenced from Supplementary Table S4: R, resistant, I intermediate, S, sensitive.

Clustering of the antibiotic resistance profiles revealed that the *P. aeruginosa* clinical strains could primarily be categorized into three branches. The upper-left cluster (6/26) exhibited resistance to β-lactam antibiotics but sensitivity to quinolones and aminoglycoside antibiotics. The middle cluster (10/26) showed overall sensitivity to antibiotics, closely resembling the WT PAO1. The lower-right cluster (10/26) exhibited significantly higher antibiotic resistance, presenting a multi-antibiotic resistant profile to cephalosporins, carbapenems, and monobactam antibiotics (Figure 2). After calculating the MAR index, 26 strains were categorized into low MAR and high MAR groups (Figure 2). It's important to note that the classification of low MAR level and high MAR level was determined based on the median MAR index, where MAR values below 0.3 were considered low MAR, and values equal to or above 0.3 were considered high MAR. Heterogeneous resistance profiles were detected in the subgroups of *P. aeruginosa* within patients B, F, G, P, and J, where both low MAR and high MAR isolates coexisted. For instance, isolate B3 from patient B, with a MAR index of 0.2, was classified as low MAR, whereas B1, with a MAR index of 0.6, was classified as high MAR, displaying higher resistance profiles (Figure 2). Similar to the phenotypes of clinical *P. aeruginosa* strains, these findings highlight the considerable complexity in antibiotic susceptibilities among *P. aeruginosa* isolates from COPD airways.

### QS phenotypes are correlated with antibiotic resistance

**Figure 3:**
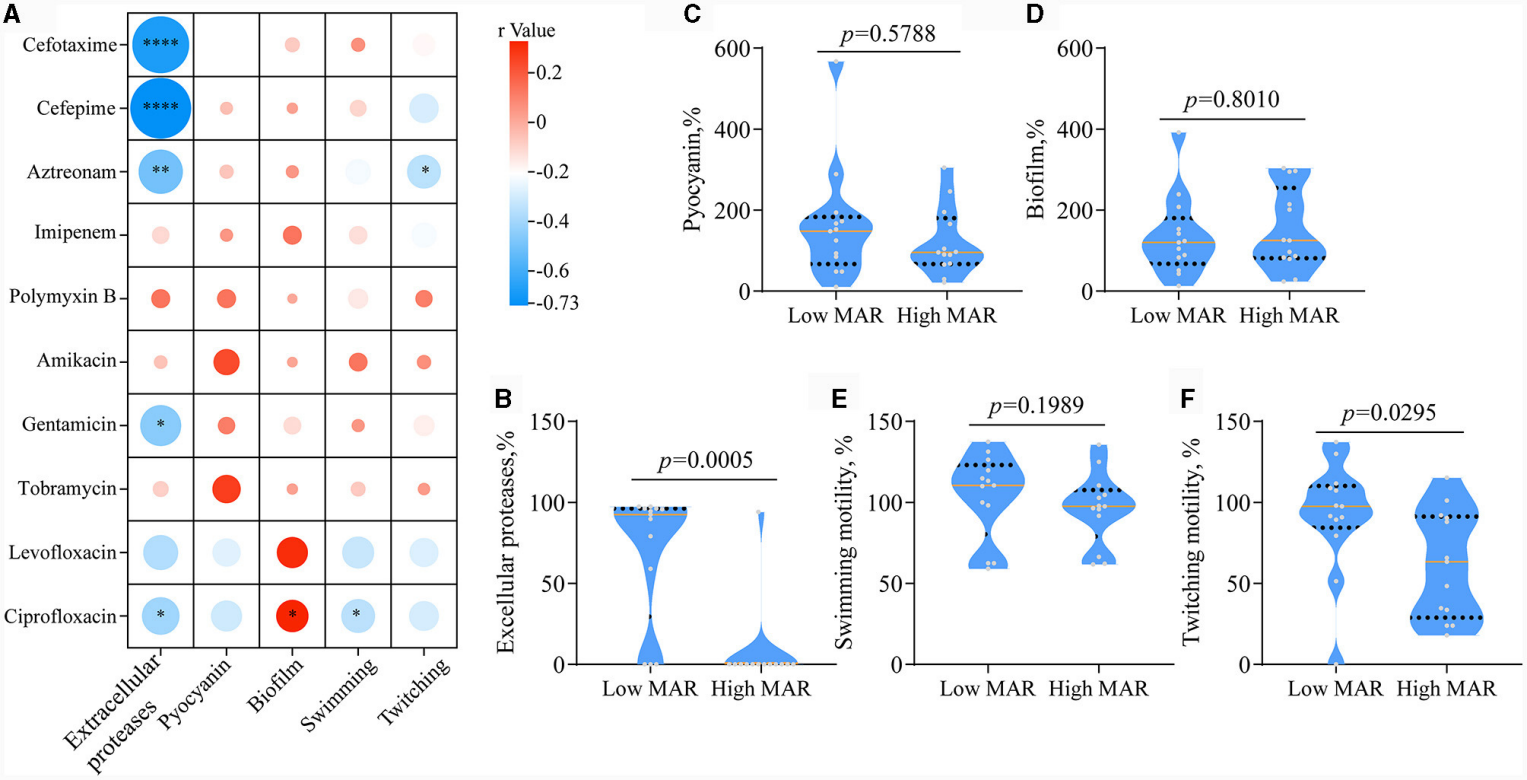
Analysis of the correlation between antibiotic resistance and QS-related virulence phenotypes. **(A)** Spearman's Rank Correlation Coefficient between minimum inhibitory concentration and quantitative phenotype. The code classifies correlation coefficients into four distinct categories based on their absolute values: WEAK (0.1–0.3), moderate (0.3–0.6), strong (0.6–0.8), and very strong (0.8–1.0). Phenotypes with weak or stronger correlations to drug resistance are highlighted with red boxes. ^*^p < 0.05, ^**^p < 0.01, and ^***^p < 0.0001. **(B–F)** Box plots comparing Extracellular Proteases, Pyocyanin, Biofilm Production, Swimming Motility, and Twitching Motility between the low MAR group (*n* = 13) and the high MAR group (*n* = 13). Unpaired two-tailed *t*-test.

We then set out to investigate whether there were characteristic phenotypes indicative of drug resistance levels in clinical *P. aeruginosa*. Upon combining the quantified heatmap of phenotypes with ERIC-PCR typing (Figures 1A, 2), It was evident that most of the strains exhibiting a high MAR profile had lost their ability to produce extracellular proteases. Conversely, strains with a low MAR profile, except for L2, H5, and G2, were proficient in secreting extracellular proteases. Spearman rank correlation coefficient analysis was performed to assess the relationship between various quantitative virulence-related phenotypes and antibiotic resistance (Figure 3A). The results showed a significant negative correlation between extracellular protease production and antibiotic resistance, with major antibiotics including Gentamicin, Cefotaxime, Levofloxacin, and Aztreonam. Biofilm production showed a positive correlation with Ciprofloxacin resistances. Twitching motility exhibited a weak negative correlation with Aztreonam resistance (Figure 3A). Among several virulence phenotypes, only the production of extracellular proteases and twitching motility showed a significant difference between the high MAR and low MAR groups (Figures 3B–F).

### The integrity of the *lasR* gene might be correlated with antibiotic resistance

**Figure 4:**
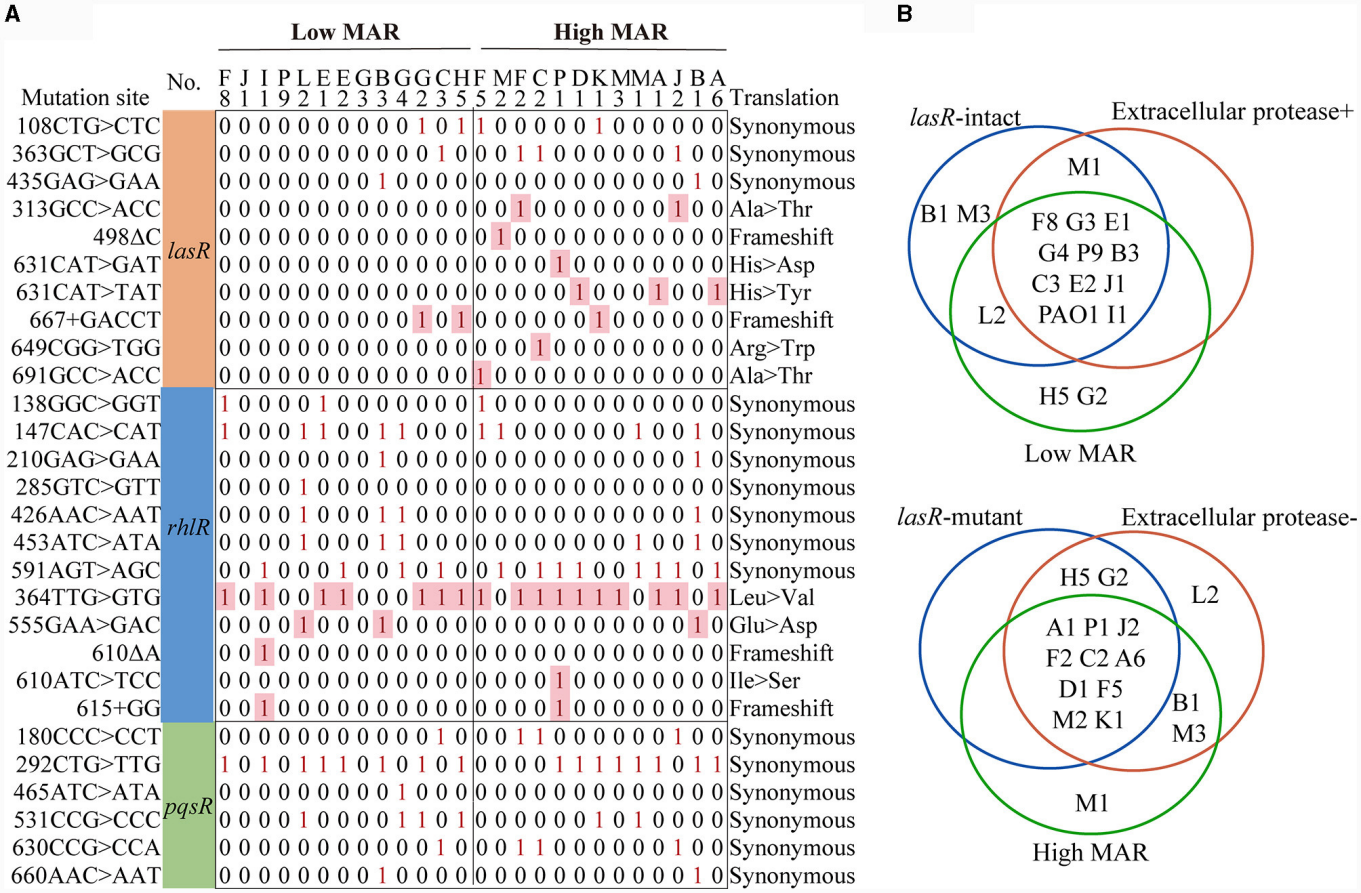
Multidrug resistance is consistently linked to *lasR* mutations and reduced extracellular protease production. **(A)** Matrix of *lasR, rhlR, pqsR* mutation sites. The sequencing results were compared with PAO1 in The *Pseudomonas* Genome Database. The matrix number 0 represents that no mutation was detected at this site, and 1 represents that mutation was detected at this site. **(B)** The Venn diagrams illustrate the classification of strains based on *lasR* integrity, MAR index, and extracellular protease characteristics.

We then checked the integrity of QS regulatory genes in *P. aeruginosa* clinical strains to explore their potential connection to antibiotic resistance development. Among the 26 clinical strains of *P. aeruginosa*, we identified six synonymous base substitution mutations in the *pqsR* gene, 12 mutation sites, including five nonsynonymous mutations, in the *rhlR* gene, and 11 mutations, of which seven were nonsynonymous, in the *lasR* gene (Figure 4A and Table 1). Additionally, 5 synonymous mutations and no nonsynonymous mutations were detected in the *lasI* gene, and 13 mutations were detected in the *rhlI* gene, two of which were nonsynonymous mutations leading to amino acid substitutions, including 122AGG>AAG (F8), 184AGC>GGC (B3, C2, C3, G4, H5, J2, M1, K1, L2; Supplementary Table S5). In summary, nonsynonymous mutations primarily occurred in the *lasR, rhlR*, and *rhlI* genes, potentially influencing the hierarchy and regulatory capacity of the QS system, and no nonsynonymous mutations were detected in the *pqsR* and *lasI* genes. Moreover, 10 out of 14 *P. aeruginosa* COPD strains with intact *lasR* gene were found to be extracellular protease-positive (10 out of 11) and low MAR (10 out of 13), as well as the reference strain PAO1. On the other hand, 10 out of 12 *P. aeruginosa* COPD strains with mutated *lasR* gene were found to be extracellular protease-negative (10 out of 15) and high MAR (10 out of 13; Figure 4B). Such kind of gene integrity and antibiotic resistance nesting was not detected in *rhlR* and *pqsR* genes. These results suggested that the correlation between the QS regulatory genes and antibiotic resistance might be established by *lasR* gene integrity.

**Table 1:** Summary of *lasR* variants with nonsynonymous mutations from COPD patients.

**Type of mutation**	**Mutation site**	**Protein translation results**
Mutant 1	G → A (+313)	105: A → T
Mutant 2	ΔC (+498)	Fragment truncation
Mutant 3	C → T (+631)	211: H → Y
Mutant 4	C → G (+631)	211: H → D
Mutant 5	+GACCT (+667)	Fragment truncation
Mutant 6	C → T (+649)	217: R → W
Mutant 7	G → A (+691)	231: A → T

### Molecular docking-based structural and functional analyses of LasR mutants

**Figure 5:**
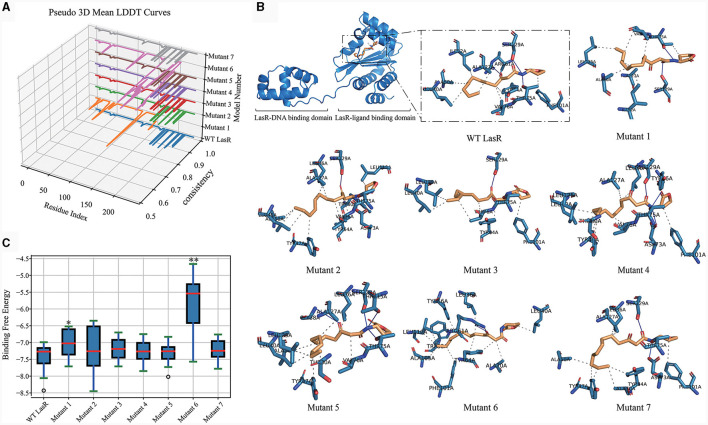
LasR Mutant Homology Modeling and Docking Analysis. **(A)** Comparative Pseudo-3D LDDT curves illustrating the quality of models generated for WT LasR and seven LasR mutants (1–7). They are performed using the Swiss-Model platform (https://swissmodel.expasy.org/) with Python, Matplotlib, and Pandas. The numbering corresponds to the mutation positions listed in Table 1. **(B)** Optimal binding of OHN (3-oxo-C12-HSL) ligand, compared for all mutants, accomplished using Dockey v0.8.2. Solid lines represent hydrogen bonds, while dashed lines represent hydrophobic or other interactions. **(C)** Box plot illustrating the distribution of binding free energies from 20 docking runs for WT and LasR mutants. *t*-test, *p < 0.05, **p < 0.01.

We then evaluated the impact of mutation types on the function of the LasR protein by using homology modeling to predict the structural alterations in different LasR mutants. Subsequently, molecular docking was conducted to predict the potential effects of these mutations on the interactions of LasR with autoinducing small molecules. The crystal structure of the LasR protein complexed with *N*-(3-oxo-dodecanoyl)-L-homoserine lactone (3-oxo-C12-HSL) was resolved, revealing its dual binding domains: a hydrophobic pocket for self-induced small molecules and an effector region binding to the deep groove of DNA (Bottomley et al., 2007) (Figure 5). Nonsynonymous mutations occurred in both distinct regions of *lasR* gene (Figure 4 and Table 1). The results of homology modeling indicated that LasR Mutant 2 and Mutant 6 displayed the most significant structural deviations from WT LasR due to structural deletions and frameshift mutations, as evidenced by the Local Distance Difference Test (LDDT) analysis. In contrast, the remaining mutants mainly represented point mutation models, demonstrating structural similarities to WT LasR (Figure 5A).

Molecular docking analysis further revealed variations in the number of hydrogen bonds and other interaction forces between autoinducing ligands and different mutant models. For instance, Mutant 1 exhibited fewer hydrogen bonds compared to WT LasR (Figure 5B), possibly due to its mutation site at position 105, strategically located within the hydrophobic pocket center, where mutations exert a more pronounced impact on the docking interactions (Table 1). Mutant 6, characterized by a fragment deletion, demonstrated a more substantial effect on the binding of small molecules and ligands, while Mutant 2 displayed an impact that, though notable, lacked statistical significance. The remaining point mutants at the DNA binding sites exhibited binding free energies similar to WT LasR or slightly higher on average but lacked statistical significance (Figure 5C).

### The virulence of *lasR* variants is multifaceted compared to WT PAO1

**Figure 6:**
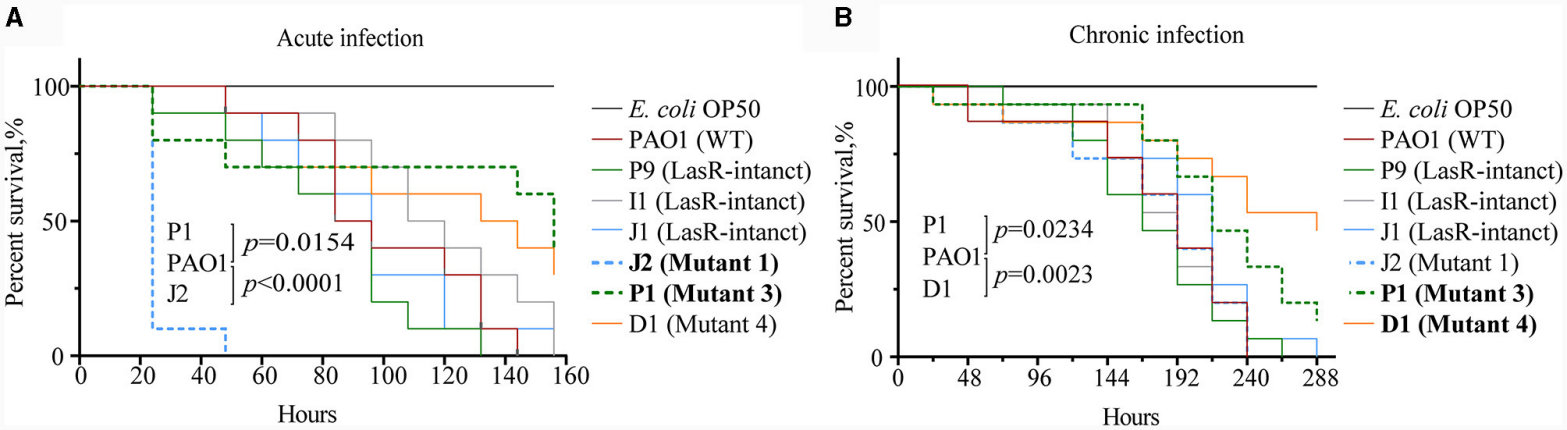
Differential virulence of *Pseudomonas aeruginosa* clinical strains with and without *lasR* mutations. **(A)** Survival curve of *Caenorhabditis elegans* with acute infection of *P. aeruginosa*. Acute infection model with three replicates per group, each containing 10 nematodes. Mantel-Cox test. **(B)** Survival curve of *C. elegans* with chronic infection of *P. aeruginosa*. Chronic infection model with three replicates per group, each containing 15 nematodes. Code labels with significant differences are bolded. Mantel-Cox test.

To validate the virulence differences between *P. aeruginosa* strains carrying *lasR* mutation and those without, this study employed *C. elegans* as an infection model and tested the fast-killing and slow-killing capabilities of six *P. aeruginosa* clinical strains. Among the tested strains, namely J1, I1, and P9, possessed intact *lasR* genes and exhibited a virulence factor expression profile similar to that of the reference strain WT PAO1. Strains J2 (mutant 1), D1 (mutant 4), and P1 (mutant 3) harbored point mutations in their *lasR* genes and failed to produce extracellular proteases (Figures 2, 4). Compared to WT PAO1, J2 (mutant 1) displayed significantly increased lethality in the fast-killing assay, resulting in complete nematode mortality 2 days post-inoculation. In contrast, P1 (mutant 3) exhibited lower virulence compared to WT PAO1, while D1 (mutant 4), I1, P9, and J1 displayed virulence levels similar to that of WT PAO1, consistent with previous virulence phenotype results (Figure 6A). In the context of the slow-killing model, D1 (mutant 4) and P1 (mutant 3) exhibited the weakest capabilities of killing nematodes, while the remaining strains, including J2 (mutant 1), showed a nematode-killing ability similar to WT PAO1 (Figure 6B). These results suggested that mutations in the *lasR* gene would influence the pathogenicity of *P. aeruginosa*, while the enhanced virulence of J2 (mutant 1) in the fast-killing assay might be attributed to the accumulation of unknown mutations in other genes during the evolution of this *lasR* mutant in COPD airway, as reported in our prior work (Zhao et al., 2023). The pathogenic mechanism of *P. aeruginosa* J2 will be explored in our further study.

### Levofloxacin and polymyxin B promote the emergence of extracellular protease-deficient strains

**Figure 7:**
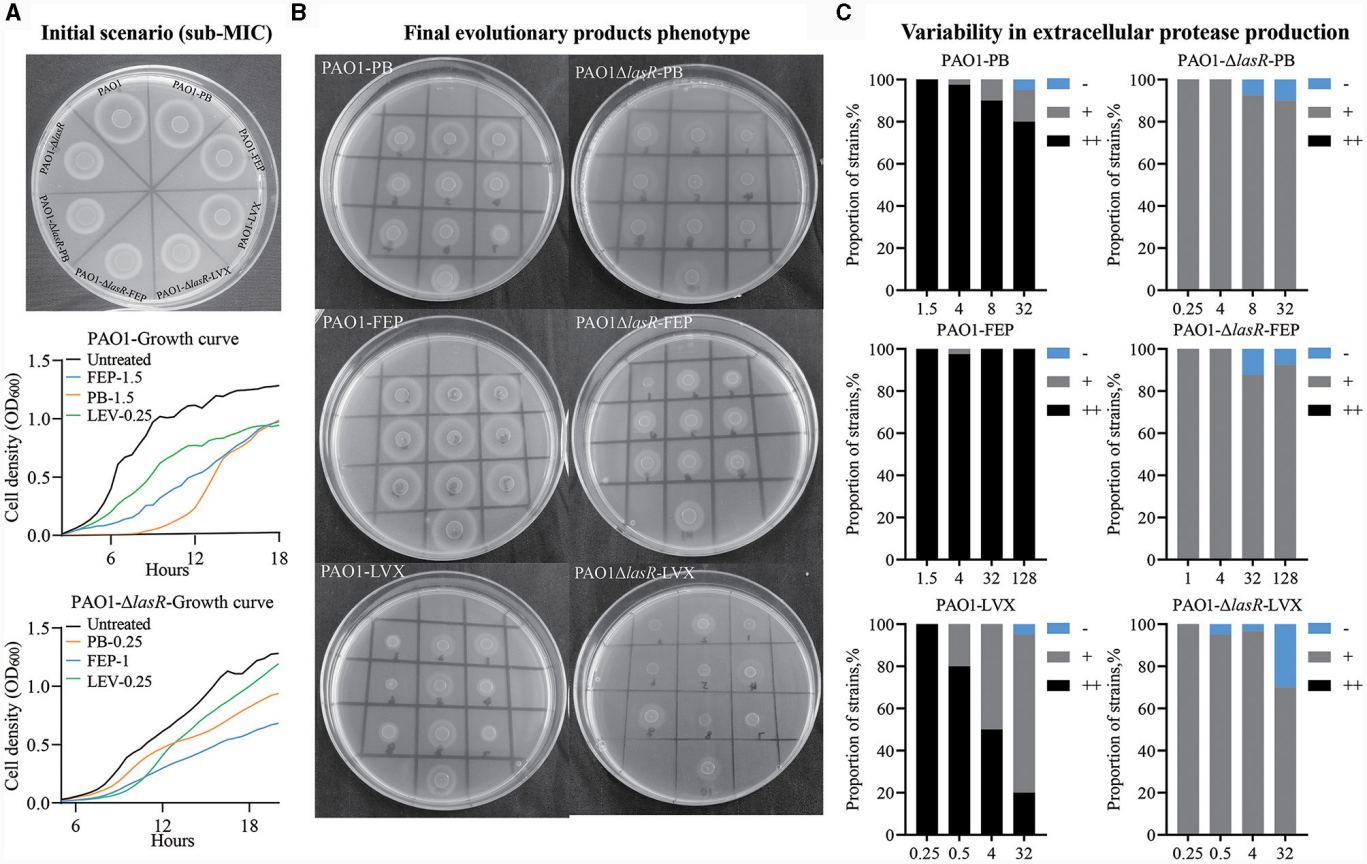
Long-term evolution of WT PAO1 and PAO1-Δ*lasR* under antibiotic pressure. **(A)** Extracellular protease production and growth curves were evaluated upon the initial application of three antibiotics at varying concentrations to PAO1 and PAO1-Δ*lasR*. **(B)** The production of extracellular protease in the final evolutionary products of each group. **(C)** Bar charts presenting the composition and statistical analysis of extracellular protease ring formation in 100 isolates. Comparison was made with the protein ring diameter of PAO1, denoted as “++” for consistency, “+” for smaller, and “–” for no protein ring production compared to PAO1. The unit of antibiotic concentration was μg/ml PB refers to Polymyxin B, FEP refers to Cefepime, and LVX refers to Levofloxacin.

To assess the adaptive influence of *P. aeruginosa* under antibiotic stress, we employed WT PAO1 and PAO1-Δ*lasR* as the initial bacterial strains and separately treated them with Levofloxacin, Cefepime, and Polymyxin B. PAO1-Δ*lasR* showed a reduction in producing extracellular proteases compared to WT PAO1 (Figure 7A). WT PAO1 reached a growth plateau at ~12 h, whereas PAO1-Δ*lasR* required around 20 h to reach a similar plateau at the same initial inoculum. Under the initial antibiotic concentrations, the addition of Levofloxacin, Cefepime, or Polymyxin B did not affect extracellular protease production in WT PAO1 and PAO1-Δ*lasR*.

Following 1 month of evolutionary culture without antibiotic treatment, the ability of WT PAO1 and PAO1-Δ*lasR* to produce extracellular proteases remained unchanged. The WT PAO1 population treated with Cefepime did not exhibit a notable reduction in extracellular protease expression, while the PAO1-Δ*lasR* group exhibited a low proportion of strains that did not produce extracellular protease. Heterogeneity was observed in both WT PAO1 and PAO1-Δ*lasR* populations after polymyxin B treatment, particularly in WT PAO1. The most pronounced heterogeneity was observed after exposure to Levofloxacin in both cases (Figures 7B, C). However, when we examined the *lasR* gene in the final evolved products of the WT PAO1 population, we detected significant A-to-G mutations at position 632 in the Levofloxacin treatment group, resulting in a histidine-to-arginine mutation at position 211 of the LasR protein. Coincidentally, clinical isolates P1, D1, A1, and A6 were found to exhibit mutations at this specific site (Figure 4). These results demonstrated that different classes of antibiotics might impose different selective pressures on the development of *P. aeruginosa* QS system. Levofloxacin and Polymyxin B, especially the former, could promote the emergence of extracellular protease-deficient strains.

## Discussion

MDR *P. aeruginosa* remains a clinical challenge, necessitating vigilant strain monitoring and precise antibiotic management for effective resistance control. *Pseudomonas aeruginosa* exhibits intrinsic resistance to multiple antibiotics, while concurrently utilizing QS systems to mediate biofilm formation (García-Contreras et al., 2015). This coupled with mechanisms such as mutations in target sites, alterations in membrane permeability, and antibiotic efflux pumps, enhances its tolerance to antibiotics, ultimately leading to the emergence of MDR *P. aeruginosa* (Hancock and Speert, 2000; Langendonk et al., 2021; Elbehiry et al., 2022). This study primarily discusses the development of antibiotic resistance and its connection to QS in *P. aeruginosa*, particularly focusing on commonly used respiratory anti-infective antibiotics.

The results of antibiotic resistance typing and phenotypic identification in this study reflect the diversity and complexity of clinical *P. aeruginosa* strains sourced from the respiratory tract of COPD patients (Figure 1). Previous studies have shown that *P. aeruginosa* undergoes differentiation into low-virulence subpopulations, to produce specific virulence factors like extracellular proteases and pyocyanin during prolonged colonization (Marvig et al., 2015; Zhao et al., 2020, 2023). However, the significant differences in the production levels of virulence factors in high MAR and low MAR *P. aeruginosa* strains, highlight the prevalence of heterogeneous resistance resulting from multiclonal subpopulation colonization and monoclonal adaptation in COPD respiratory infections. Strains with negative extracellular protease typically exhibit multidrug resistance characteristics. Additionally, these strains often harbor mutations in the *lasR* gene. Conversely, strains with positive extracellular protease usually demonstrate lower antibiotic resistance and possess intact *lasR* genes (Figures 2–4). This also suggests a parallel trend between *lasR* mutations and the rise in antibiotic resistance (Oshri et al., 2018; Azimi et al., 2020).

In the *in vitro* evolution experiments, the present study demonstrated that different antibiotics might impose diverse influences on the development of *P. aeruginosa* QS system. This can be supported by the remarkably increased frequencies of extracellular protease-deficient strains in Polymyxin B- or Levofloxacin-treated WT PAO1 and the *lasR* mutant identified from Levofloxacin-treated WT PAO1 (Figure 7). Additionally, antibiotic is the sole factor that brings the main selective pressure during the evolution of *P. aeruginosa* in LB medium containing an antibiotic. However, the PAO1-Δ*lasR* strain used in this study showed no increase in antibiotic resistance, and its resistance to Polymyxin B was even decreased compared to the parental WT PAO1 (Supplementary Table S2). Therefore, it is hard to simply conclude that antibiotics exert selective pressure on *lasR* gene or that *lasR* mutations facilitate the development of resistance. It is also important to note that the production of extracellular proteases by *P. aeruginosa* is not exclusively governed by the *lasR* gene. Hence, while antibiotic exposure may select for extracellular protease-deficient phenotype, the direct causation by *lasR* remains uncertain. The indirect contribution of antibiotic-driven selective pressure on the utilization of sharable extracellular products to the emergence of *lasR*-deficient strains (Oshri et al., 2018), causing diversity in the QS system integrity within the population, still necessitates further validation.

In conclusion, this study dissects the negative correlation between antibiotic resistance and QS system in clinical *P. aeruginosa*, establishing a connection mediated by extracellular protease-deficient phenotypes. While adaptation theory generally suggests that *P. aeruginosa* in chronic infections may evolve toward decreasing QS regulation and virulence (Jiricny et al., 2014), our results indicate that the QS mutants, especially the strains deficient in producing extracellular proteases, exhibit increasing levels of resistance and present distinct risks in comparison to QS-intact strains. These findings also provide an important reference for further research and development of novel antimicrobial agents, and for the selection and application of clinical antibiotics to treat *pseudomonal* infections.

## Data availability statement

The raw data supporting the conclusions of this article will be made available by the authors, without undue reservation.

## Ethics statement

The studies involving humans were approved by Affiliated Hospital of Chengdu University. The studies were conducted in accordance with the local legislation and institutional requirements. The participants provided their written informed consent to participate in this study.

## Author contributions

XY: Investigation, Methodology, Writing – original draft. QZ: Investigation, Project administration, Resources, Writing – review & editing. SG: Investigation, Writing – review & editing. YW: Investigation, Writing – review & editing. XM: Investigation, Writing – review & editing. HZ: Investigation, Resources, Writing – review & editing. KZ: Conceptualization, Data curation, Formal analysis, Funding acquisition, Methodology, Project administration, Supervision, Validation, Writing – review & editing, Writing – original draft.
