# Supplementary material for: Phenotypic heterogeneity unveils a negative correlation between antibiotic resistance and quorum sensing in Pseudomonas aeruginosa clinical isolates
Source: Front Microbiol. 2024 Feb 12;15:1327675. doi: 10.3389/fmicb.2024.1327675 (PMC10895058; doi:10.3389/fmicb.2024.1327675)
Supplement: Supplementary file 1 [file Data_Sheet_1.PDF]

Phenotypic Heterogeneity Unveils a Negative Correlation Between Antibiotic Resistance and Quorum Sensing in *Pseudomonas aeruginosa* Clinical Isolates

Xiting Yang[1], Qianglin Zeng[2], Shiyi Gou[1], Yi Wu[1], Xiaoling Ma[1], Hang Zou[1], Kelei Zhao[1,*]

[1] Antibiotics Research and Re-evaluation Key Laboratory of Sichuan Province, School of Pharmacy, Chengdu University, Chengdu 610106, Sichuan, China.

[2] Affiliated Hospital of Chengdu University, Chengdu University, Chengdu 610081, Sichuan, China.

*** Correspondence:**

Kelei Zhao, address: No. 2025, Chengluo Avenue, Chengdu 610106, Sichuan, China. Tel.: +86–028–84216035. Email: zhaokelei@cdu.edu.cn.

This file contains Supplementary Figure S1 and Tables S1 to S4.

Supplementary Figures

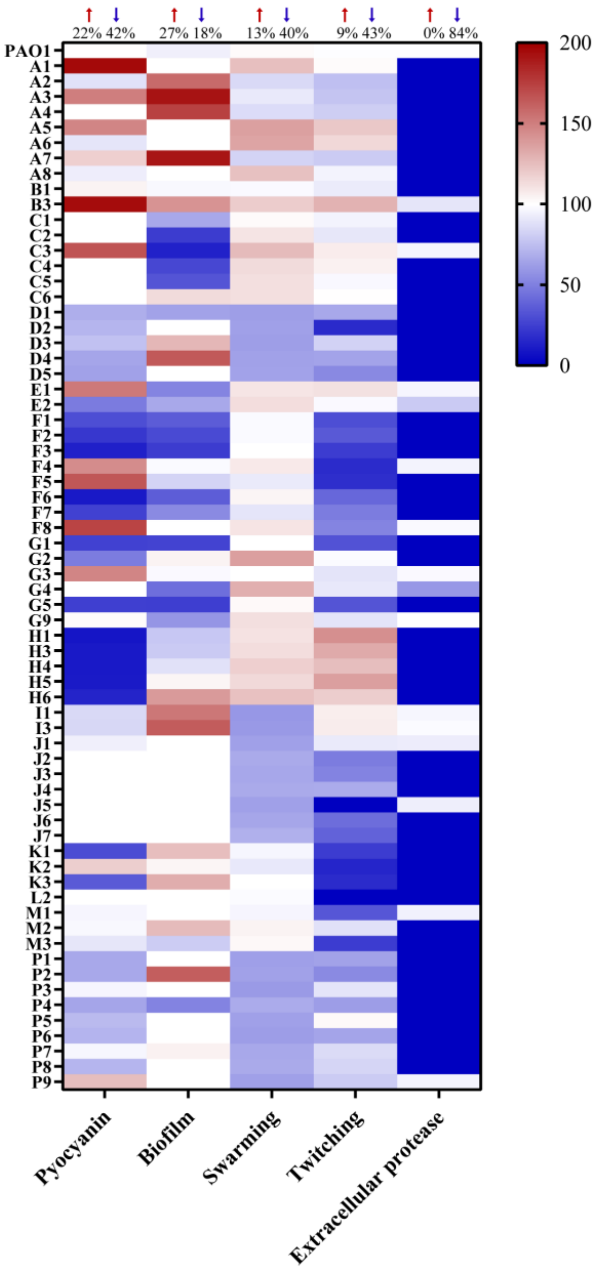

Supplementary Figure 1. Heatmap depicting the quantitative phenotypic variations related to quorum sensing system regulation in 67 clinical bacterial strains compared to PAO1. The red arrow points to the proportion of upregulated strains for this phenotype, while the blue arrow points to the proportion of downregulated strains for this phenotype. Data are mean, n = 3 per group.

Supplementary Tables

Supplementary Table S1. Patient code and sampling information.

Patient code	Age	Gender	Disease	Use of antibiotics during hospitalization
A	83	male	AECOPD, Bacterial pneumonia	Cephalosporins, penicillins, quinolones
B	63	male	AECOPD, Severe pneumonia	Cephalosporins, quinolones, carbapenems
C	62	male	AECOPD, Severe pneumonia	Penicillins, quinolones
D	81	male	COPD, Severe pneumonia	Cephalosporins, quinolones, carbapenems, aminoglycosides
E	73	male	AECOPD	Cephalosporins, quinolones, carbapenems, aminoglycosides
F	92	male	AECOPD, Severe pneumonia	Penicillins, glycopeptides, carbapenems
G	82	male	AECOPD, Severe pneumonia	Cephalosporins, penicillins, quinolones
H	74	male	COPD, Severe pneumonia	Cephalosporins, penicillins, quinolones
I	70	male	COPD, Severe pneumonia	Quinolones, glycopeptides, cephalosporins
J	65	male	AECOPD, Bacterial pneumonia	Penicillins, glycopeptides
K	60	male	COPD, Severe pneumonia	Carbapenems, cephalosporins, quinolones
L	75	male	AECOPD, Severe pneumonia	Cephalosporins, penicillins
M	84	female	COPD, Severe pneumonia	Penicillins, quinolones, carbapenems
P	81	male	COPD, Severe pneumonia	Aminoglycosides, quinolones, carbapenems

Note: AECOPD, Acute Exacerbation of Chronic Obstructive Pulmonary Disease.

Supplementary Table S2. The MICs of Polymyxin B, Cefepime, and Levofloxacin for WT PAO1 and PAO1-Δ*lasR*.

	Polymyxin B	Cefepime	Levofloxacin
PAO1	2 μg/mL	2 μg/mL	0.5 μg/mL
PAO1-Δ*lasR*	0.5 μg/mL	2 μg/mL	0.5 μg/mL

Supplementary Table S3. The concentrations of antibiotics at different days in the in vitro evolution assays.

	PAO1			PAO1-Δ*lasR*		
Time	PB	FEP	LVX	PB	FEP	LVX
Day 1	1.5	1.5	0.25	0.25	1	0.25
Day 4	2	2	0.5	0.5	2	0.5
Day 8	4	4	1	1	4	1
Day 12	8	8	2	2	8	2
Day 16	16	16	4	4	16	4
Day 20	32	32	8	8	32	8
Day 24		64	16	16	64	16
Day 28		128	32	32	128	32

Note: Antibiotic units are µg/mL. PB refers to Polymyxin B, FEP refers to Cefepime, and LVX refers to Levofloxacin.

Supplementary Table S4. Susceptibility testing of *P. aeruginosa* clinical strains to 10 antibiotics.

	CLSI	Resistant(R)		Intermediate(I)		Sensitive(S)	
Antibiotics	Quality Control Range	Interpretation Range	Strains	Interpretation Range	Strains	Interpretation Range	Strains
Aztreonam	2-8	≤64	5	≤32	8	≤8	13
Cefotaxime	8-32	≤256	12	≤64	2	≤32	12
Cefepime	0.5-4	≤32	3	≤8	5	≤4	18
Imipenem	1-4	≤32	9	≤8	5	≤4	12
Polymyxin B	0.5-2	≤64	0	≤16	4	≤2	22
Levofloxacin	0.5-4	≤64	0	≤16	2	≤4	24
Ciprofloxacin	0.25-1	≤16	0	≤4	8	≤1	18
Amikacin	1-4	≤64	0	≤16	1	≤4	25
Gentamicin	0.5-2	≤32	0	≤8	0	≤2	26
Tobramycin	0.25-1	≤16	1	≤4	1	≤1	24

Note: Antibiotic units are μg/mL.

Supplementary Table S5. Summary *lasI* and *rhlI* variants from COPD patients.

Gene	Mutation site	Protein translation results
lasI	G→A (+48)	16: L→L
	T→C (+150)	50: S→S
	C→T (+432)	144: T→T
	C→G (+441)	147: G→G
	G→T (+531)	177: R→R
rhlI	G→A (+78)	26: Q→Q
	C→T (+105)	35: D→D
	A→G (+138)	46: E→E
	C→T (+195)	65: G→G
	C→T (+207)	69: C→C
	C→T (+217)	73: L→L
	G→C (+282)	94: T→T
	C→A (+297)	99: V→V
	C→T (+312)	104: R→R
	G→A (+327)	109: A→A
	G→A (+342)	114: Q→Q
	G→A (+122)	41: R→K
	A→G (+184)	62: S→G
